# Adherent invasive *Escherichia coli* (AIEC) strain LF82, but not *Candida albicans*, plays a profibrogenic role in the intestine

**DOI:** 10.1186/s13099-021-00401-z

**Published:** 2021-01-28

**Authors:** Dina Chokr, Marjorie Cornu, Christel Neut, Clovis Bortolus, Rogatien Charlet, Pierre Desreumaux, Silvia Speca, Boualem Sendid

**Affiliations:** 1 Univ. Lille, Inserm, CHU Lille, U995 - LIRIC - Lille Inflammation Research International Centre, Team Fungal Associated Invasive & Inflammatory Diseases, 59000 Lille, France; 2 Laboratoire de Parasitologie Mycologie, CHU Lille, Univ. Lille, 59000 Lille, France; 3 Univ. Lille, Inserm, CHU Lille, U995 - LIRIC - Lille Inflammation Research International Centre, Team Inflammatory Digestive Diseases: Pathophysiology and Therapeutic Targets Development, 59000 Lille, France; 4 Faculté de Médecine - Pôle Recherche, Place Verdun, 59045 Lille Cedex, France; 5 Present Address: Inserm U1285, UMR CNRS 8576- UGSF, Villeneuve d’Ascq, France

**Keywords:** Inflammatory bowel disease, Intestinal fibrosis, TGF-β-stimulated intestinal epithelial cells, AIEC strain LF82, *C. albicans*

## Abstract

**Background:**

Intestinal fibrosis is a frequent complication of Crohn’s disease. However, the factors that cause chronicity and promote fibrogenesis are not yet understood.

**Aims:**

In the present study, we evaluated the profibrotic effects of adherent-invasive *Escherichia coli* (AIEC) LF82 strain and *Candida albicans* in the gut.

**Methods:**

Colonic fibrosis was induced in C57BL/6 mice by administration of three cycles of 2.5% (w/v) dextran sulfate sodium (DSS) for 5 weeks. LF82 and *C. albicans* were administered orally once at the start of each week or each cycle, respectively. Expression of markers of myofibroblast activation was determined in TGF-β1-stimulated human intestinal epithelial cells (IECs).

**Results:**

LF82 administration exacerbated fibrosis in DSS-treated mice, revealed by increased colonic collagen deposition and expression of the profibrotic genes *Col1a1*, *Col3a1*, *Fn1* and *Vim*. This was accompanied by enhanced gene expression of proinflammatory cytokines and chemokines, as well as more recruited inflammatory cells into the intestine. LF82 also potentiated TGF-β1-stimulated epithelial–mesenchymal transition and myofibroblast activation in IECs, by further inducing gene expression of the main mesenchymal cell markers *FN1* and *VIM* and downregulating the IEC marker *OCLN*. Proinflammatory cytokines were overexpressed with LF82 in TGF-β1-stimulated IECs. Conversely, *C. albicans* did not affect intestinal fibrosis progression in DSS-treated mice or myofibroblast activation in TGF-β1-stimulated IECs.

**Conclusions:**

These results demonstrate that AIEC strain LF82, but not *C. albicans*, may play a major profibrogenic role in the gut.

## Introduction

Crohn’s disease (CD) is a worldwide chronic inflammatory bowel disease (IBD) whose incidence is increasing across Europe [1–3]. Although the precise aetiology of CD is unknown, it is well accepted that this disease is the consequence of immune-mediated injury to the gut mucosa inflicted by an overactive immune response towards environmental factors in a genetically predisposed host [4]. Sustained inflammation and chronic wound healing response often lead to intestinal fibrosis, a condition defined by an excessive accumulation of extracellular matrix (ECM) proteins produced by activated myofibroblasts, which are alpha smooth muscle actin (α-SMA)-expressing cells. These cells not only derive from resident mesenchymal cells (fibroblasts and smooth muscle cells) but can also originate from epithelial and endothelial cells via epithelial/endothelial transition, as well as from stellate cells, pericytes and bone marrow stem cells [5]. Activation of myofibroblasts occurs in response to different stimuli, including growth factors such as transforming growth factor beta (TGF-β) and platelet-derived growth factor (PDGF), pro-inflammatory cytokines such as interleukin 1 (IL-1), IL-13 and IL-17, as well as CC chemokines like CCL2, CCL3 and CCL4, and lipid mediators released by immune and non-immune cells [6]. As a result, the formation of fibrotic scars in the intestinal wall results in a narrowing of the intestinal lumen and generates strictures and fistulae, or stenosis, in approximately 50% of CD patients. Despite the availability of treatments that target inflammation, no effective anti-fibrotic therapies exist, with surgical intervention being the only curative option although inflammation and stenosis may reoccur [5, 7, 8]. There is therefore an urgent need for a better understanding of the pathophysiology and identification of potential therapeutic targets in intestinal fibrosis.

A growing body of evidence suggests that an imbalance in the gut microbiota, or dysbiosis, is highly associated with CD pathogenesis, where it modulates the inflammatory status [9]. The bacterial microbiome has also been linked to intestinal fibrosis; however, the direct correlation between specific microbial species and fibrogenesis is still uncertain. CD patients are characterized by having fewer bacterial phyla, *Firmicutes* and *Bacteroidetes*, with anti-inflammatory properties, and more *Actinobacteria* and *Proteobacteria*, with proinflammatory roles [9, 10]. Among the Gram-negative *Proteobacteria*, pathogenic adherent*-*invasive *Escherichia coli* (AIEC) has been preferentially observed in ileal CD [11, 12]. The prototype AIEC strain, LF82, colonizes the intestinal mucosa and induces proinflammatory cytokines in a number of acute dextran sulfate sodium (DSS)-induced colitis mouse models [13–15]. Adhesion to intestinal epithelial cells (IECs) has been shown to be mediated by the interaction of LF82 type 1 pili with the abnormally expressed human carcinoembryonic antigen-related cell adhesion molecule 6 (CEACAM6) [14], the binding of flagella to TLR5 and IPAF flagellin receptors [13], or via chitin-binding domains, encoded by bacterial chitinase ChiA, that interact with human chitinase CHI3L1 expressed on IECs under inflammatory conditions [15].

In addition to bacteria, fungal microbiota dysbiosis is also considered a possible cause of CD. A number of studies have shown a decrease in levels of non-pathogenic *Saccharomyces cerevisiae* yeasts [16, 17] and an increase in abundance of *Candida* species in CD patients [18]. *Candida albicans* is the most prevalent opportunistic fungal pathogen in the intestine. Our group has previously demonstrated that colonic inflammation induced by DSS promotes *C. albicans* colonization in mice. In turn, *C. albicans*, via the β-galactoside-binding lectin receptor galectin-3, augments inflammation, as revealed by increased expression of TLR-2 and TNF-α, and triggers antibody generation directed against *C. albicans* antigens and also anti-*S. cerevisiae* antibodies (ASCA), which are serological markers of CD [19, 20]. Together, these findings indicate that microorganisms and their products can be profibrogenic in the gut.

In the current study, we investigated the effects of the AIEC strain LF82 and *C. albicans* on chronic intestinal inflammation and fibrosis progression.

## Results

### LF82, but not *C. albicans*, exacerbated intestinal fibrosis in DSS-induced chronic colitis in mice

**Fig. 1:**
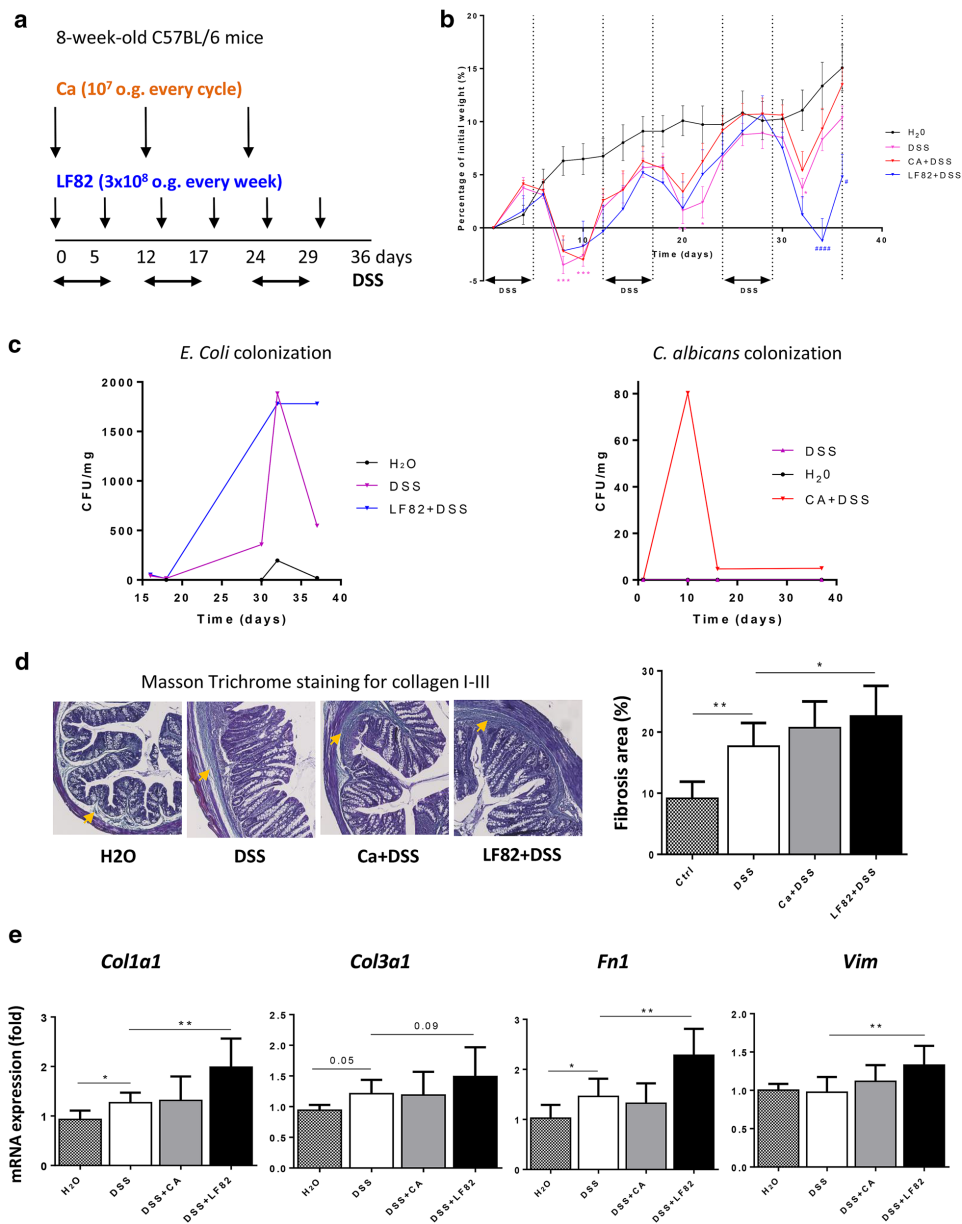
Chronic AIEC LF82, but not *C. albicans*, administration worsened intestinal fibrosis in DSS-induced chronic colitis in mice. **a** Illustration of the experimental design of DSS-induced chronic colitis. Wild-type mice (n = 5–10 mice/group) received 2.5% DSS in drinking water in three cycles (5 days of DSS followed by 7 days of drinking water) and were orally administered LF82 (3 × 10^8^) or *C. albicans* (10^7^) once at the beginning of each week or each cycle, respectively. **b** Body weight change (percentage of initial weight) of different groups (from week 0 to week 5). Data are means ± SEM; *P < 0.05, **P < 0.01, ***P < 0.001 for DSS-exposed mice vs. mice receiving drinking water; ^#^*P* < 0.05, ^####^*P* < 0.0001 for colonized vs. non-colonized mice receiving DSS (two-way ANOVA test with a Bonferroni post hoc correction). **c** Quantification of *E. coli* or *C. albicans* (CFU/mg) in culture of faeces recovered from mouse groups on different days. **d** Representative images and quantification of Masson’s trichrome staining of colonic tissue sections. Orange arrows indicate collagen deposition (green) in the colonic submucosa and mucosa. **e** Colonic gene expression of *Col1a1*, *Col3a1*, *Fn1* and *Vim*. Results correspond to fold-increase when compared to mice receiving drinking water and are expressed as means ± SEM; *P < 0.05; **P < 0.01 for colonized vs. non-colonized mice receiving DSS (Mann–Whitney U test)

To examine the profibrotic role of the AIEC strain LF82 and *C. albicans* on the intestinal tract, chronic colitis was induced in mice exposed to 3 cycles of DSS as described previously [21]. The experimental design is illustrated in Fig. 1a. A previous study reported that wild-type (WT) mice challenged orally with LF82 showed neither colonization nor gut inflammation after 7 days [14]. Based on those results, LF82 was administered once per week to DSS-treated mice; *E. coli* counts in the stools increased in LF82-colonized DSS mice and persisted for 36 days (Fig. 1c). *C. albicans* administration was initiated once per cycle in DSS-treated mice according to a study done by our group showing colonization and persistence of *C. albicans* in the intestine of mice after acute DSS exposure for 7 days [20]. Our study demonstrated evidence of *C. albicans* colonization in the first cycle of DSS administration which persisted until the end of the experiment (Fig. 1c). As expected, mice treated with DSS showed decreased body weight during all cycles compared to control mice (H_2_O treated animals) (Fig. 1b). DSS-exposed mice colonized with LF82 showed significantly greater weight loss during the last DSS cycle when compared to DSS-treated mice (Fig. 1b).

Intestinal fibrosis was assessed by histological analysis using Masson trichrome staining of collagen I–III within the colon tissue. Chronic DSS exposure caused a statistically significant increase in collagen I–III deposition in the colon subepithelium and serosal areas, an effect that was further enhanced by the presence of LF82 only (Fig. 1d). Moreover, quantitative RT-PCR performed on entire colon samples, showed that the expression of the profibrotic genes *Col1a1*, *Col3a1* and *Fn1* was elevated with DSS exposure. LF82, but not *C. albicans* administration, was able to further amplify the expression of these three profibrotic genes, in addition to *Vim*, in DSS-treated mice (Fig. 1e). These results suggest that the severity of fibrogenesis was affected by LF82 but not by *C. albicans* presence in the DSS-induced model of chronic colitis.

### LF82 and *C. albicans* differentially exhibit proinflammatory properties in DSS-induced chronic colitis in mice

**Fig. 2:**
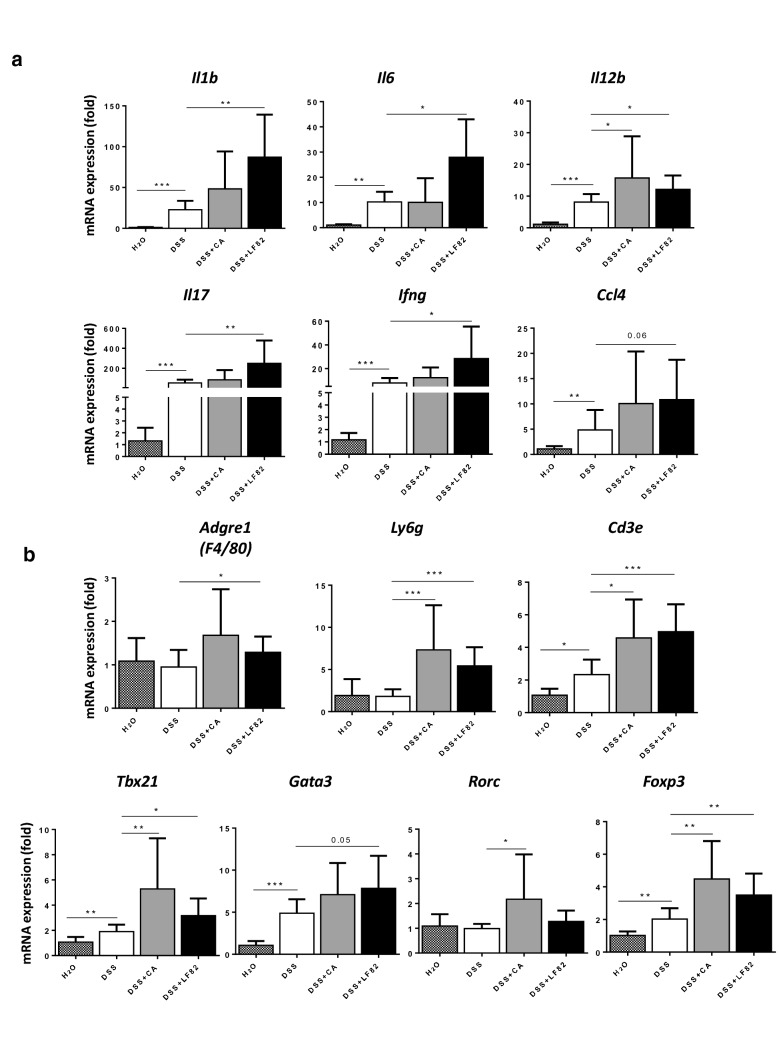
Chronic AIEC LF82 and *C. albicans* administration induced intestinal inflammation in DSS-induced chronic colitis in mice. Colonic gene expression of: **a** proinflammatory cytokines *Il1b*, *Il6*, *Il12b*, *Il17*, *Ifng* and chemokine *Ccl4*; **b** immune cell recruitment markers *Adgre1*, *Ly6g* and *Cd3e*, and T-cell transcription factors *Tbx21*, *Gata3*, *Rorc* and *Foxp3.* Results correspond to fold-increase when compared to mice receiving drinking water and are expressed as means ± SEM; *P < 0.05; **P < 0.01; ***P < 0.001 for colonized vs. non-colonized mice receiving DSS only (Mann–Whitney U test)

Fibrosis is known to require chronic inflammation as a prerequisite. Thus, we analysed the impact of LF82 and *C. albicans* on the gene expression of colonic proinflammatory cytokines and chemokines. Colonic expression of the genes *Il1b*, *Il6*, *Il12b*, *Il17*, *Ifng* and *Ccl4* was increased in the presence of DSS and overexpressed in the presence of LF82 (Fig. 2a). No significant differences in mRNA levels of most cytokines and chemokines were detected except for *Il12b* in *C. albicans*-colonized mice compared to uninfected controls after DSS exposure (Fig. 2a). We also examined the consequence of these two microorganisms on recruitment of different inflammatory cells into the intestine. DSS was able to induce the expression of the T-cell marker *Cd3e* and, in particular, Th1-specific *Tbx21/Tbet*, Th2-specific *Gata3* and Treg-specific *Foxp3* transcription factors (Fig. 2b). LF82 led to a significant further increase in gene expression of the macrophage/monocyte marker *Adgre1-F4/80*, the neutrophil marker *Ly6g*, *Cd3e*, *Txb21*, *Gata3* and *Foxp3* in DSS-treated groups, while *C. albicans* showed highly elevated mRNA levels of *Ly6g*, *Cd3e*, *Txb21*, Th17-specific *Rorc/Rorγt* transcription factor and *Foxp3* in DSS-exposed mice (Fig. 2b). These data imply that whereas only LF82 worsened fibrogenesis, both LF82 and *C. albicans* were able to differently induce intestinal inflammation.

### LF82, but not *C. albicans*, enhanced TGFβ-induced epithelial–mesenchymal transition (EMT) and myofibroblast activation of human epithelial cells

**Fig. 3:**
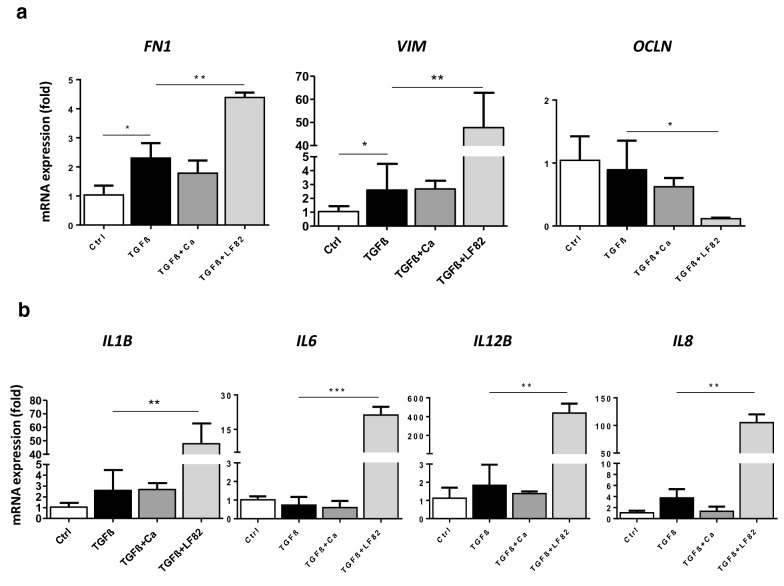
LF82, but not *C. albicans*, potentiated epithelial–mesenchymal transition (EMT) and myofibroblast activation of monolayers of human intestinal Caco-2 cells stimulated by TGF-β for 4 days. **a** Only LF82 induced overexpression of mesenchymal cell markers *FN1* and *VIM*, and downregulation of the IEC marker *OCLN* in TGF-β-stimulated Caco-2 cells. **b** LF82 also increased the expression of the proinflammatory cytokines *IL1B*, *IL6*, *IL12B* and *IL8* after TGF-β stimulation. Experiments were performed in triplicate and data correspond to fold-increase compared to control mice. Data are expressed as means ± SEM; *P < 0.05; **P < 0.01; ***P < 0.001 (Student’s t-test). Results are representative of three independent experiments

We then performed an in vitro experiment to determine the effect of LF82 and *C. albicans* on myofibroblast differentiation of monolayers of human IECs in the Caco-2 cell line in the presence of TGF-β1. Optimal TGF-β1 administration conditions inducing the differentiation of IECs to a myofibroblast phenotype were standardized. Four days of 20 ng/mL TGF-β1 exposure was able to significantly increase the expression of myofibroblast and activation markers, *FN1* and *VIM*, without affecting the expression of the IEC marker *OCLN* or proinflammatory cytokines *IL1B, IL6*, *IL12B* and *IL8* (Fig. 3). TGF-β1-stimulated Caco-2 cells challenged with LF82 presented significant overexpression of *FN1* and *VIM* and reduced gene expression of *OCLN*, while there was no effect on expression of these genes when they were challenged with *C. albicans* (Fig. 3a). Moreover, only LF82 highly increased the gene expression of *IL1b*, *IL6*, *IL12b* and *IL8* after TGF-β exposure (Fig. 3b). These results indicate that LF82, but not *C. albicans*, potentiated myofibroblast activation of IECs by EMT induced by TGF-β1.

## Discussion

Intestinal fibrosis is the most common complication in patients with CD. It is the final outcome of the gut mucosal reaction to chronic inflammation and repair, which results in excessive deposition of extracellular matrix, leading eventually to intestinal dysfunction. Despite substantial efforts to identify the triggers for intestinal inflammation, the mechanisms underlying fibrosis remain poorly characterized and delay the development of effective anti-fibrotic therapies. Mounting evidence indicates that microorganisms can correlate directly with intestinal inflammation and could predict fibrosis. Here, we identified the AIEC strain LF82 as a new candidate organism affecting intestinal fibrogenesis, while the fungus *C. albicans* had no profibrogenic effect.

Most previous studies have shown that alterations in the bacterial microbiome are implicated in intestinal fibrosis. Antibiotic treatment in rats with chronic colitis significantly prevented TGFβ-1, collagen production and stricture formation [22], and intramural injection of faecal material or extracts from anaerobic bacteria into the intestinal wall induced chronic colitis with fibrosis and elevated levels of TGFβ-1 in colonic tissue [22, 23]. Additionally, a recent study showed that *Tl1a*-overexpressing mice had reduced colonic collagen deposition under pathogen-free conditions and interestingly proved causality by demonstrating that specific bacteria or bacterial consortia including groups of mucolytic bacteria such as *Mucispirillum schaedleri*, *Ruminococcus*, *Anaeroplasma* and members of the *Streptococcus* and *Lactobacillus* genera are directly correlated with the degree of fibrosis and fibroblast phenotype [24]. Severe and persistent intestinal fibrosis also occurred in mice infected chronically with *Salmonella enterica* [25].

AIEC is an *E. coli* pathotype that is present in high numbers in the inflamed gut of CD patients and its role in chronic colitis-associated fibrosis was first demonstrated in a chronic infection model of fibrosis using the AIEC strain NRG857c. NRG857c colonization persists for months in WT mice and is accompanied by chronic transmural inflammation and fibrosis [26]. This model was used in an attempt to avoid the use of LF82, which does not colonize conventional mice beyond 7 days and requires the expression of human CEACAM6 receptors to develop intestinal inflammation after acute DSS exposure [14]. In the present study, we observed the effects of LF82 on intestinal inflammation and fibrosis in WT mice in response to chronic DSS exposure by repeated LF82 challenges every 7 days. We demonstrated that LF82 worsened fibrosis, revealed by increased collagen deposition in the colon subepithelium and serosal areas and enhanced expression of the main fibrotic genes *Col1a1*, *Col3a1*, *Fn1* and *Vim*.

Inflammation in CD has been shown to be driven predominantly by Th1 and Th17 responses [27]. We observed high gene expression of *Il17*, as well as of *Ifng*, which was consistent with elevated mRNA expression of the Th1 transcription factor *Tbet*, suggesting a Th1 response in our model. Other experimental colitis models have shown that both Th1- and Th2-mediated pathways contribute to the pathogenesis of CD, where they participate in different stages of chronic colitis development and affect diverse components of the inflammatory response [28, 29]. This may explain the increase in the Th2 transcription factor *Gata3* in LF82-challenged mice after DSS exposure. Mucosal Treg cells and activated macrophages are also increased in paediatric and adult CD patients [30]. In our system, the Treg transcription factor *Foxp3* was expressed at increased levels*.* Furthermore, there was also an upregulation in gene expression of the monocyte/macrophage marker *Adgre1* and the neutrophil marker *Ly6g*, as well as higher expression levels of the proinflammatory cytokines related to them such as *Il1b*, *Il6* and *Il12b*. Our results are similar to those observed with chronic NRG857c inflammation involving Th1 and Th17 responses and a significant role for macrophages and Treg cells [26].

Since EMT has been identified as a key contributor to the pool of activated fibroblasts associated with fibrosis in a mouse model of chronic colitis and to fistula formation in CD patients [31, 32], we evaluated the in vitro effect of LF82 on EMT and myofibroblast activation in TGF-β1-stimulated human IEC Caco-2 cells. LF82 aggravated TGF-β1-stimulated myofibroblast activation of these cells by EMT, as revealed by highly increased gene expression of mesenchymal cell markers *FN1* and *VIM* and downregulated expression of the IEC marker *OCLN*. TGF-β signalling in epithelial cells appears to play an anti-inflammatory role [33]; however, we did not observe any effect of TGF-β on expression of the proinflammatory cytokines *IL1B*, *IL6*, *IL12B* and *IL8* in our model. LF82 had strong proinflammatory properties and thus was able to overexpress all of these genes in IECs in the presence of TGF-β. Overall, these findings indicate that LF82 is associated with severe intestinal inflammation and fibrosis, and can affect fibroblast function directly or possibly via its products. Future studies should identify the mechanism for LF82-stimulated intestinal inflammation and fibrosis.

The importance of the fungal microbiome has received little attention in the context of intestinal fibrosis. Here, we evaluated whether *C. albicans*, the most prevalent fungal species in CD patients, is positively or negatively correlated with fibrosis severity. Despite the pro-inflammatory effects observed, we found that *C. albicans* did not affect fibrosis severity in DSS-treated mice. This could be due to certain products of this fungus that may have opposing effects on intestinal inflammation compared to fibrosis and this requires further investigation. In vitro studies showed no impact of *C. albicans* on myofibroblast activation and proinflammatory properties of TGF-β1-stimulated IECs, thus confirming the lack of a profibrogenic effect of *C. albicans* and that its effect on inflammation depends on different cell types.

Epithelial integrity is compromised in DSS-induced colitis suggesting penetration of microbes and diffusion of associated antigens into the mucosa [34]. One could thus think that the observed effects in our experiments had nothing to do with LF82 itself but reflect a difference between yeasts and bacteria, or may be attributed to both LF82 and other microbes colonizing the gut including other *E. coli* strains. Pathogenic and commensal *E. coli* were both increased in CD [35], but there was no direct evidence that they could directly affect intestinal fibrosis. However, it has been shown that TLR4, which could be activated by Gram-negative derived LPS, mediates chronic intestinal inflammation and fibrosis by regulating cytokine expression on intestinal macrophages and myofibroblasts and inducing epithelial–mesenchymal transition [36]. These findings suggest a role for *E. coli* in inducing colitis and fibrosis. Additionally, it has been reported that stimulation of intestinal myofibroblasts with LPS can upregulate TLRs (2, 3, 4, 6, 7) and their accessory molecules (MyD88, TIRAP), activate the MAPK pathway and increase IL-8 secretion, thus indicating that intestinal myofibroblasts participate in the immune response in the intestine after activation by bacterial products and may play a role in CD-associated fibrosis [37]. In our study, the effects of DSS on gut epithelial injury, resulting in increased intestinal permeability, changes in the microbiota and immunological and fibrotic alterations, as observed in the DSS-treated group, were in the same range as in the LF82-treated and *C. albicans-*treated DSS-induced groups. On the other hand, LF82 enhanced fibrogenesis compared to the DSS-treated group, meaning that we had an additive or synergistic proinflammatory and profibrogenic role of LF82. In addition, in in vitro experiments, LF82 enhanced the effect of the fibrogenic cytokine TGFβ inducing EMT and myofibroblast activation.

## Conclusions

In conclusion, this study demonstrates a role for LF82 AIEC strains in the intestinal fibrotic process and suggests a possible role of the gut microbiome in the evolution of intestinal inflammation. To our knowledge, this is the first study to address the involvement of the fungal microbiome in intestinal fibrosis, ruling out a positive correlation between *Candida* species and fibrosis progression in our model.

## Materials and methods

### Strains and cultures of bacteria and yeasts

The AIEC strain LF82, derived from a chronic ileal lesion in a CD patient, was kindly provided by Christel Neut (Faculté de Médecine, Pôle Recherche, Laboratoire J&K, Lille, France) and maintained on MacConkey agar. *C. albicans* was isolated from the stool of a CD patient and maintained on Sabouraud dextrose agar containing amikacin. Prior to the experiments, LF82 and *C. albicans* were incubated overnight on a shaker at 37 °C in Luria–Bertani broth and yeast peptone dextrose broth, respectively, and harvested by centrifugation for 3 min at 3000×*g* for bacteria and 500×*g* for yeasts. The pellets were resuspended in 1× phosphate*-*buffered saline (PBS).

### Animals

A total of 41, 7–8-week-old, male, C57/BL6 mice obtained from Janvier Laboratories (France) were used in the study and housed under specific pathogen-free conditions.

### Mouse model of DSS-induced chronic colitis and fibrosis and infection

Chronic colitis and fibrosis were induced in mice by oral administration of 2.5% (w/v) DSS (MW: 36,000–44,000, purchased from TdB Consultancy, Uppsala, Sweden) in drinking water and administered ad libitum for three cycles (5 days of DSS followed by 7 days of tap water), as described previously [21]. Mice were challenged orally with 200 µL of PBS containing 3 × 10^8^ LF82 live cells once per week, or with 200 µL of PBS containing 10^7^ *C. albicans* live cells once per cycle. Mice were distributed into two control groups, including mice receiving drinking water (H_2_O) (n = 5) or DSS (n = 10) alone, and two experimental groups (n = 9–10/group), including LF82 + DSS and *C. albicans* (CA) + DSS. Animals underwent regular clinical follow-up (stool consistency and bleeding) and were weighed at the beginning of the study and every 2 days thereafter. Following oral administration, *E. coli* and *C. albicans* colonization in the intestinal tract was monitored by counting the number of colony-forming units (CFUs) in faeces collected from different animals. Faecal homogenates were plated on the corresponding agars (as mentioned above) and incubated at 37 °C for 24 h to 1 week. Blood and colon tissues were collected at sacrifice.

### Cell culture and treatment

The human IEC line Caco-2 (ATCC HTB-37) was grown in Dulbecco’s modified Eagle's medium (DMEM) supplemented with 100 U/mL penicillin, 100 μg/mL streptomycin and 10% foetal bovine serum (FBS). Cell cultures were maintained in a humidified atmosphere of 95% air/5% CO_2_ at 37 °C. Cells were seeded in 12-well plates at a density of 2.5 × 10^5^ and incubated in medium without serum and supplemented with 20 ng/mL TGF-β1 for 4 days for myofibroblast activation. Cells were treated with LF82 and *C. albicans* at a multiplicity of infection = 1 during the last 24 h.

### Masson trichrome collagen staining

Colon specimens from all animals were fixed overnight in 4% paraformaldehyde-acid, embedded in paraffin, cut to a thickness of 4 μm and stained with modified Masson’s trichrome counterstain using a Tissue-Tek Prisma Automated Slide Stainer (Sakura). The reagents for this staining technique include: haematoxylin, Mallory red (acid fuchsin stain and orange G), phosphotungstic–phosphomolybdic acid solution and light green, and when applied sequentially the resulting stain colours are: nuclei—dark blue, cytoplasm—pink, erythrocytes—bright red, muscles and collagen—green. The slides were scanned with a ZEISS Axio Scan and images were analysed using ImageJ software (NIH, USA).

### RNA isolation and real time-PCR

Total RNA from entire mouse colon samples was isolated using Trizol reagent (Thermo Fisher Scientific) and RNA from human intestinal cell lines was extracted with a NucleoSpin RNA II kit (Macherey–Nagel) following the manufacturer’s instructions. Genomic DNA was removed using DNase I (RNase-free) according to the manufacturer’s protocol. RNA quantification was performed by spectrophotometry (Nanodrop). Reverse transcription was carried out on 1 µg RNA using a High Capacity cDNA reverse transcriptase kit (Thermo Fisher Scientific) and real time-PCR was performed with Fast SYBER Green Master Green (Thermo Fisher Scientific) according to the manufacturer's protocol. Gene expression from entire mouse colon samples and human IECs was normalized to *Polr2a* and *GAPDH*, respectively. The sequences and relative house-keeping genes are listed in Table 1.

**Table 1:** Sequences and house-keeping genes

Primers	Source	Forward sequence	Reverse sequence
Col1a1	Mouse	GAGTACTGGATCGACCCTAACCAA	ACACAGGTCTGACCTGTCTCCAT
Col3a1	Mouse	GCCCACAGCCTTCTACAC	CCAGGGTCACCATTTCTC
Fn1	Mouse	CGAAGCCGGGAAGAGCAAG	CGTTCCCACTGCTGATTTATCTG
Vim	Mouse	CCGTTCAAGGTCAAGACGTGCC	AGGAGGCCGAAAGCACCCTGC
Il1b	Mouse	AGCTCTCCACCTCAATGGAC	AGGCCACAGGTATTTTGTCG
Il6	Mouse	TACACATGTTCTCTGGGAAATCGT	AAGTGCATCATCGTTGTTCATACA
Il12b	Mouse	GGAAGCACGGCAGCAGAAT	GGCGGGTCTGGTTTGATG
Il17a	Mouse	GCAAGAGATCCTGGTCCTGA	AGCATCTTCTCGACCCTGAA
Ifng	Mouse	CAGCAACAGCAAGGCGAAA	CTGGACCTGTGGGTTGTTGAC
Ccl4	Mouse	CCACTTCCTGCTGTTTCTCT	TTGGTCAGGAATACCACAGC
Adgre1	Mouse	CTTTGGCTATGGGCTTCCAGTC	GCAAGGAGGACAGAGTTTATCGTG
Ly6g	Mouse	TGGACTCTCACAGAAGCAAAG	GCAGAGGTCTTCCTTCCAACA
Cd3e	Mouse	ATGCGGTGGAACACTTTCTGG	GCACGTCAACTCTACACTGGT
Tbx21	Mouse	TTCCCATTCCTGTCCTTCAC	CCACATCCACAAACATCCTG
Gata3	Mouse	GGAAACTCCGTCAGGGCTA	AGAGATCCGTGCAGCAGAG
Rorc	Mouse	GAAAGCAGGAGCAATGGAAG	GATGGAAAGCCAGTTCCAAA
Polr2a	Mouse	GGTGCTGTGGGTACGGATACA	CCCACAACCAGCTATCCTCAA
FN1	Human	GATGCTCCCACTAACCTCCA	CGGTCAGTCGGTATCCTGTT
VIM	Human	GCAGGCTCAGATTCAGGAACA	GTGAGGTCAGGCTTGGAAACA
OCLN	Human	AGGACGTGCCTTCACCCCCA	ACCACCGCTGCTGTAACGAGG
IL1B	Human	GATGCACCTGTACGATCACT	GACATGGAGAACACCACTTG
IL6	Human	AGTGAGGAACAAGCCAGAGC	GTCAGGGGTGGTTATTGCAT
IL12B	Human	CCTGACCATCCAAGTCAAAGAGT	AGGAGCGAATGGCTTAGAACCT
IL8	Human	AAATCAGGAAGGCTGCCAAGA	AAGGAACCATCTCACTGTGTGTAAAC
GAPDH	Human	GACACCCACTCCTCCACCTTT	TTGCTGTAGCCAAATTCGTTGT

### Statistical analysis

Data are expressed as the mean ± SEM. The Student’s t-test was used to compare the mean values of two related groups. The Mann–Whitney U test was used for comparisons between two independent groups. Two-way ANOVA test with a Bonferroni post hoc correction was used for multiple intergroup comparisons. The results were considered statistically significant at *P* < 0.05. All statistical analyses were performed using Prism software (Graphpad, La Jolla, CA).

## Data Availability

The datasets generated during and/or analysed during the current study are available from the corresponding authors on reasonable request.
